# Exposure to local, source-specific ambient air pollution during pregnancy and autism in children: a cohort study from southern Sweden

**DOI:** 10.1038/s41598-023-30877-5

**Published:** 2023-03-08

**Authors:** Erin Flanagan, Ebba Malmqvist, Ralf Rittner, Peik Gustafsson, Karin Källén, Anna Oudin

**Affiliations:** 1 Division of Occupational and Environmental Medicine, Department of Laboratory Medicine, Faculty of Medicine, Lund University, Lund, Sweden; 2 Child and Adolescent Psychiatry, Department of Clinical Sciences, Faculty of Medicine, Lund University, Lund, Sweden

**Keywords:** Paediatric neurological disorders, Neurodevelopmental disorders, Autism spectrum disorders, Paediatric research, Public health, Epidemiology, Environmental health

## Abstract

Evidence of air pollution exposure, namely, ambient particulate matter (PM), during pregnancy and an increased risk of autism in children is growing; however, the unique PM sources that contribute to this association are currently unknown. The aim of the present study was to investigate local, source-specific ambient PM exposure during pregnancy and its associations with childhood autism, specifically, and autism spectrum disorders (ASD) as a group. A cohort of 40,245 singleton births from 2000 to 2009 in Scania, Sweden, was combined with data on locally emitted PM with an aerodynamic diameter < 2.5 µm (PM_2.5_). A flat, two-dimensional dispersion model was used to assess local PM_2.5_ concentrations (all-source PM_2.5_, small-scale residential heating- mainly wood burning, tailpipe exhaust, and vehicle wear-and-tear) at the mother’s residential address during pregnancy. Associations were analyzed using binary logistic regression. Exposure to local PM_2.5_ during pregnancy from each of the investigated sources was associated with childhood autism in the fully adjusted models. For ASD, similar, but less pronounced, associations were found. The results add to existing evidence that exposure to air pollution during pregnancy may be associated with an increased risk of childhood autism. Further, these findings suggest that locally produced emissions from both residential wood burning and road traffic-related sources (tailpipe exhaust and vehicle wear-and-tear) contribute to this association.

## Introduction

Air pollution is a major cause of morbidity and mortality worldwide^1^. Particulate matter (PM) with an aerodynamic diameter less than 2.5 µm (PM_2.5_) derived from fossil fuels alone has recently been estimated to contribute to 10.2 million premature deaths around the world each year^2^. The relationship between traffic-related air pollution, often tailpipe emissions stemming from diesel fuel and petrol combustion, and adverse health effects is well established^3–5^. Additionally, vehicle wear-and-tear’s finer fractions contribute to inhalable PM pollution^6^. Wood burning for residential heating and leisure purposes also produces PM as well as other toxic compounds^7–9^, yet little is known about the health effects of ambient wood smoke.

Evolving evidence indicates that health effects may vary in type and degree depending on the source of PM_2.5_ exposure^10^. Source-specific epidemiological studies are still scarce, though, as such separation requires high quality exposure data that has not been available previously. Air pollution is far from static and varies substantially in time and space, which means that advanced exposure assessment is needed to better capture local exposures’ unique composition. With this, the health effects of distinct sources, rather than total concentrations, can be investigated. For instance, associations between tailpipe exhaust particles and low birth weight have been observed in a Swedish study using advanced source-specific exposure data^11^. Associations between ambient wood smoke, and their related markers, and both dementia^12^ and childhood asthma^13^ have also been found in other Sweden-based studies using advanced exposure assessment data.

While all people are susceptible to the adverse health effects of air pollution, pregnant women and their developing fetuses may be particularly vulnerable^14,15^. Thus, air pollution exposure during pregnancy and in early life has been studied in connection to autism spectrum disorders (ASD). Evidence of this association has been developing, with a 2016 systematic review and meta-analysis supporting the association but emphasizing cautious interpretation due to the relatively small sample of available studies^16^. A 2019 systematic review and meta-analysis including both adults and children has demonstrated more robust associations, naming ASD among other neurological and developmental disorders associated with PM_2.5_ exposure^17^. More current systematic reviews and meta-analyses focusing on children only and prenatal exposure specifically identified the strongest evidence for PM_2.5_ exposure and ASD^18,19^, with one reporting each 5 µg/m^3^ incremental increase in PM_2.5_ to lead to a 5%, 7%, or 15% increased risk of ASD in newborns depending on the model used^19^. Similarly, a 2022 systematic review and meta-analysis examining vulnerable exposure windows, including prenatal periods (first trimester, third trimester and entire pregnancy), first year after birth, and second year after birth, also reported an association between PM_2.5_ and ASD, and the authors named early postnatal periods as critical exposure windows^20^. Some disagreement exists, however, as another review found the pregnancy period to be the most impactful PM_2.5_ exposure window for ASD^21^.

Autism development is poorly understood, but mounting evidence has emphasized the importance of genetic and environmental factors^22^. With the latter being modifiable, research on the role of the environment, particularly air pollution, has increased. Suggested biological mechanisms include oxidative stress, inflammation (neuro-inflammation and systemic inflammation), neurotoxicity, and endocrine disruption^23–26^. Animal models also support developmental neurotoxicity as a plausible pathway^27,28^, and one such study has demonstrated ASD traits in mice following prenatal exposure to diesel exhaust^29^. A recent study of maternal serum samples indicated that high exposure to traffic-related air pollution during pregnancy disturbed metabolic pathways and mitochondrial function among mothers with children that developed ASD^30^. Moreover, short- and long-term species of PM_2.5_ that can be traced to wood burning have been shown to negatively affect several metabolic pathways involved in oxidative stress and inflammation^31^.

Although air pollution has been demonstrated to significantly affect neurodevelopment and contribute to autism^18,19^, only one study could be identified considering these associations with respect to source-specific exposure (tailpipe and non-tailpipe emissions from road traffic)^32^. As the sources of locally produced air pollution may vary from setting to setting and each source can have unique effects on human health, previous research has encouraged more source-apportionment studies of PM^33^. Road traffic is often one of the largest contributors to PM emissions and is predicted to increase^19^, therefore, exploring the various sources of traffic-related PM can provide a better understanding of their individual impacts on public health. Additionally, too few epidemiological studies investigating ambient wood smoke from residential wood combustion and health exist to date^34^, and none consider autism. Indeed, a review on the health impacts of wood burning emissions cited the need for more studies on non-respiratory pediatric outcomes, with better exposure assessment and proper adjustment for confounding^35^. The knowledge gleaned from source-specific findings can be used to inform air quality policy on all potentially detrimental emission sources.

The aim of the present study was to investigate the association between exposure to source-specific ambient PM_2.5_ during pregnancy and autism in children within a low-exposure setting where air pollution concentrations generally comply with the current European air quality guidelines^36^.

## Methods

### Study setting and study population

This study was undertaken in Scania (Skåne), the southernmost county in Sweden, which had a total population of approximately 1.4 million at the end of 2019. The study population is comprised of the Maternal Air Pollution in Southern Sweden (MAPSS) cohort, containing data on 43,256 singleton births from 2000 to 2009 in Scania. A detailed description of this cohort has been provided previously by Malmqvist et al.^37^. In short, MAPSS utilizes a high-quality local birth register with 98% coverage of all births in Scania and includes important data on the women’s obstetric history, risk factors, and other relevant covariates. The birth register, Perinatal Revision Syd (PRS), has a wide catchment area of hospitals in Malmö, Lund, and Trelleborg.

**Figure 1:**
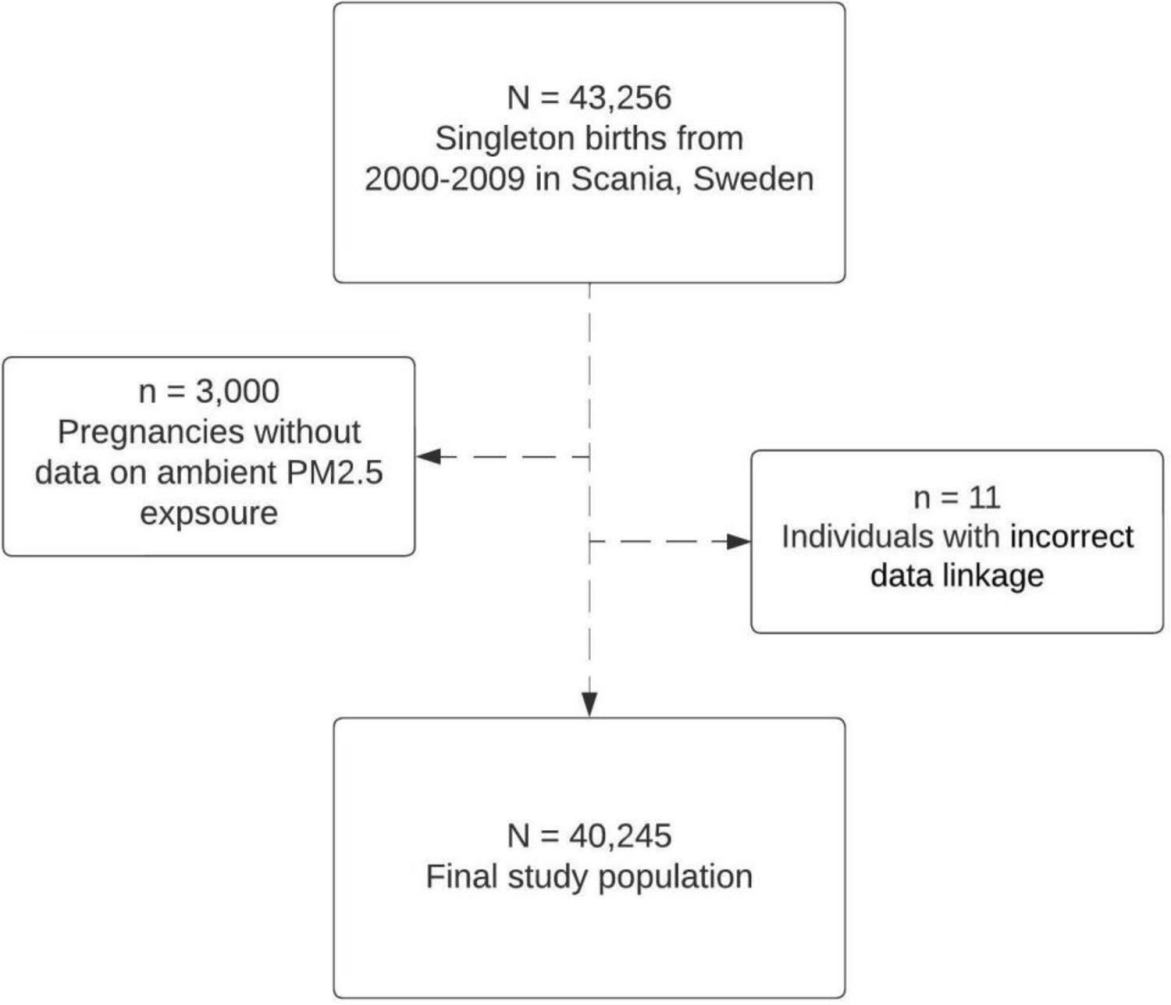
Flowchart of the study population by exclusion of individuals falling outside the study area and due to missing data or incorrect data linkage.

Using each woman’s unique personal identification number, PRS was linked to air pollution concentrations at her residential address as well as to additional demographic information and socioeconomic characteristics obtained from Statistics Sweden. This data combined constitutes MAPSS. When excluding children with missing exposure or incorrect data linkage, the final study size was 40,245 (Fig. 1).

### Exposure assessment

A Gaussian plume air dispersion model was used to model the dispersion of emissions throughout Scania, Sweden, during the study period^38^. This flat, two-dimensional dispersion model is a locally adjusted version of the American Meteorological Society/Environmental Protection Agency Regulatory Model (AERMOD) by the United States Environmental Protection Agency^39^ and was implemented in the ENVIMAN software package. Total PM_2.5_ concentrations were modelled at a 100 m by 100 m spatial resolution and a standard, hourly temporal resolution, which was then aggregated into yearly concentrations to derive annual mean concentrations. Source-specific PM_2.5_ concentrations were based on dispersion calculations utilizing statistical weather data, which consisted of 360 different conditions. These were derived from monitored weather conditions over a long period. The software calculated dispersion for each PM_2.5_ source according to each of the 360 conditions, multiplied the result for each case with the frequency in which the condition occurred during the monitored period, and summed these to obtain an annual mean concentration. Because modelling each individual 100-m grid-square throughout the entire study area (11,303 km^2^) and for the whole study period (12 years) is both resource- and time-intensive, the dispersion calculation was only carried out for the start and end years (2000 and 2011) of the study period. The interpolation of the years between them was based on an atmospheric ventilation index using a complex method developed by the Swedish Clean Air and Climate Research Program. To derive monthly values, further interpolation was performed and is described in Rittner et al.^38^. During this process, month- and year-specific temporal and meteorological variations in air pollution were addressed. The model was evaluated against measured monthly mean PM_2.5_ concentrations, aggregated from hourly or daily averages, from three monitoring stations in Malmö, Trelleborg and Landskrona (along the west and southwest coast of Scania). Correlations (Pearson R^2^) between modelled and measured PM_2.5_ concentrations ranged from 0.44 to 0.86, with the highest correlation seen in the southwest part of Scania where Malmö, the largest city in Scania, is located^38^. Mean bias of the PM_2.5_ model was negative and positive spanning: − 8.99 to 4.59 µg/m^3^, which does not indicate the presence of systematic modelling error in any specific direction^38^. The root mean square error (RMSE) for the three monitoring stations ranged between 1.50 and 9.33^38^.

Emission data for this dispersion model was obtained from a detailed database of local emission sources maintained by the City of Malmö’s Environmental Department. These included aviation, industries, major energy and heat producers, railroads, road traffic, shipping, small-scale residential heating, non-road vehicles, and emissions from Zealand, Denmark. Aviation emission data from regional airports and Kastrup (Copenhagen, Denmark) were obtained from the airports’ annual environmental reports. Industry and energy production emissions were reported by relevant entities within the study area. Railroads in Sweden are mainly electric; therefore, railroad emissions were estimated using the fuel consumption of the few operational diesel engine freight trains in Scania while in transit on railway lines and while static at railway stations^37^. To account for emissions from road traffic, data on fuel sources (petrol, diesel, and compressed natural gas), types of vehicles (passenger cars, light-goods vehicles and heavy-duty vehicles), traffic volume, and speed limits were known for each road segment (classified into one of 36 distinct road types in an urban or rural environment) in the study area. This data was gathered from the Swedish Road Administration and local municipalities. Emission factors developed by the Handbook of Emission Factors for Road Transport (HBEFA), version 3.2^40^ were then applied to this data to estimate local traffic exhaust emissions. Emissions from vehicle wear-and-tear, or the abrasive emissions from road traffic, included re-suspended road dust, tire-wear and brake-wear particles according to the emission model detailed in HBEFA 3.2^40^. Originally, only the re-suspension of PM_10_ particles was included in the emission database. To include PM_2.5_, it was estimated that only 20% of PM_10_ particles were less than 2.5 µm in diameter (i.e., PM_2.5_ equals 0.2 of PM_10_)^38^. The suspension of road dust and tire-wear during wintertime was also based on research analyzing particulate emissions from studded tires in Stockholm, Sweden. These were then applied to the Scanian setting according to an estimate of the percentage of vehicles with studded tires (35% in 2000 and 25% in 2011^38^). Shipping emissions from around Scania’s coast were estimated by Gustafsson^39^ for the year 2000, and 2011 emissions were described by Project Shipair^41^. Regarding small-scale residential heating, the frequency of, for example, fireplace- and/or wood stove-use in Scania was estimated using detailed chimney sweeping records^39^. Emissions from non-road vehicles throughout the study area were derived from a report by the Swedish Environmental Research Institute, IVL. Scania’s proximity to the industrial island of Zealand and the area’s prevailing westerly winds warranted the inclusion of their local emissions in the dispersion model^38^.

The geographical coordinates of each woman’s residential address were obtained from Statistics Sweden, linked to MAPSS, and used to calculate individual exposure. Statistics Sweden only updates changes in residency at the end of the calendar year. Exposure estimates for every gestational month were, therefore, based on the nearest available time: January–June coordinates from the end of the previous year and July–December coordinates from the end of the current year. Moreover, if 67% or more of monthly exposure data was non-missing, the pregnancy exposure overall was designated as non-missing. This cut-off was chosen following an evaluation of monthly exposures for pregnancies with complete exposure data. For pregnancies where > 67% of the monthly exposure data was missing, all missing values were replaced with an estimated value based on exposure from subsequent years using the Expectation–Maximization algorithm.

Because this study examined locally emitted PM_2.5_, regional background concentrations (often referred to as “long-range” or “in-transported”) were not considered. As regional background emissions typically comprise the majority of total PM, investigating only local PM concentrations results in seemingly low exposure levels. However, the spatial contrast of regional background concentrations is low in this study area^38^. Because of this, effect estimates would still describe contrasts in local exposures even if regional background concentrations were included. The interpretation of the estimates becomes somewhat different, though: it illustrates the effect of local contrasts rather than the aggregate effect of total PM, which is more traditionally studied.

**Figure 2:**
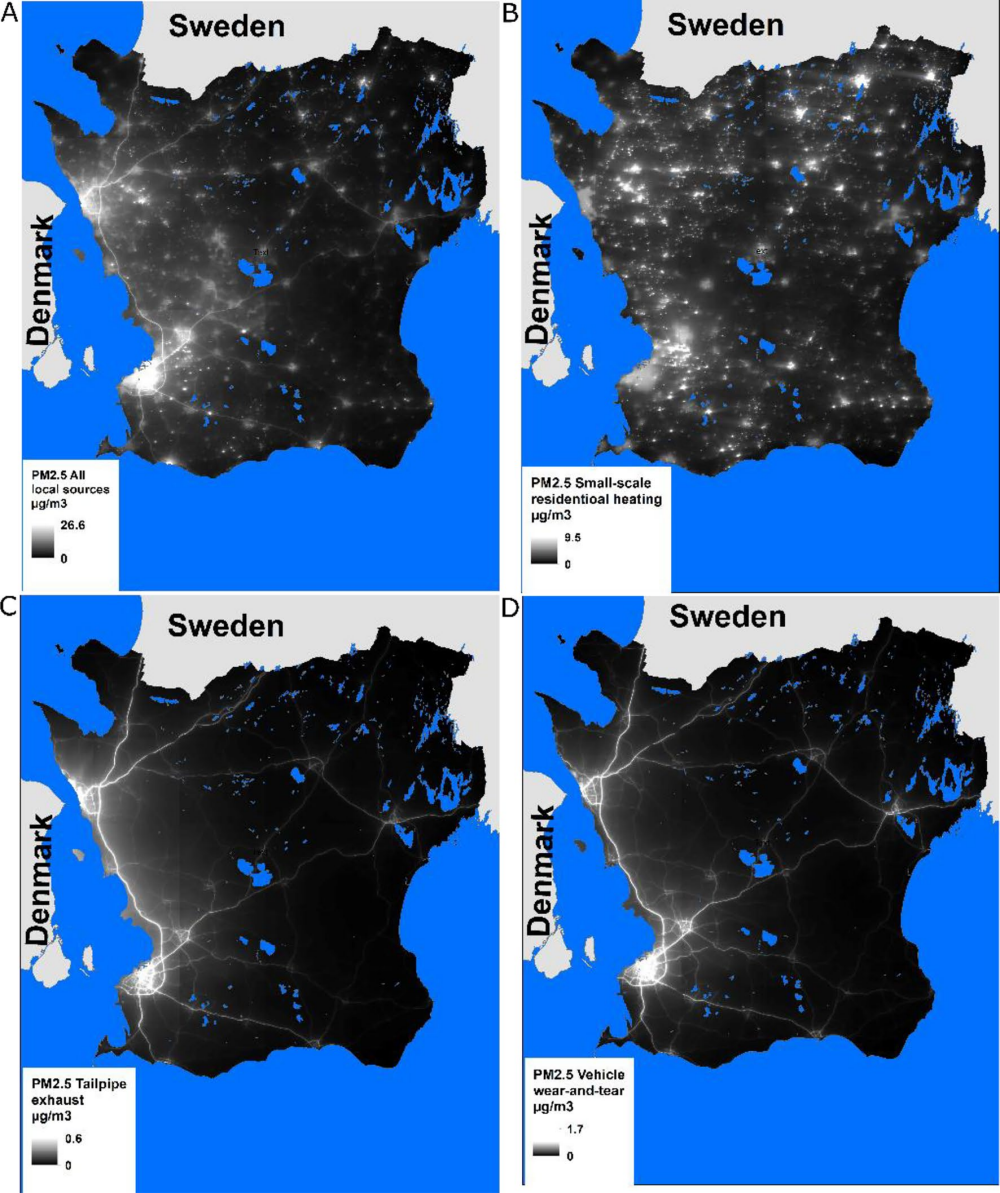
Annual mean concentrations (µg/m^3^) of locally produced PM_2.5_ from (**A**) all-source PM_2.5_, (**B**) small-scale residential heating, (**C**) tailpipe exhaust, and (**D**) vehicle wear-and-tear for the year 2009 in Scania, Sweden. The maps were created using R version 3.6 (https://www.R-project.org/) and ESRI ArcGIS version 10.5.1 (https://www.esri.com).

Four sources of locally emitted PM_2.5_ were investigated: all-source PM_2.5_, small-scale residential heating, tailpipe exhaust, and vehicle wear-and-tear. Small-scale residential heating mainly consists of wood burning in household fireplaces and/or wood stoves for heating or leisure, and vehicle wear-and-tear comprises the air pollution generated from re-suspended road dust and vehicles’ brakes and tires (Fig. 2). All other source contributions (aviation, industries, major energy and heat producers, railroads, shipping, non-road vehicles, etc.) were very small. Therefore, these were not examined separately but were captured by the aggregated all-source PM_2.5_ category, which also included small-scale residential heating, tailpipe exhaust, and vehicle wear-and-tear. The distribution of these exposure variables can be seen in Supplementary Fig. S1 (Supplementary Information).

### Outcome assessment

The outcome of interest in this study was autism, specifically ASD [International Classification of Mental and Behavioral Disorders version 10 (ICD-10) diagnosis codes starting with F84] and childhood autism (ICD-10 diagnosis code F84.0) only. ASD comprises all pervasive developmental disorders. It is characterized by one or more of the following areas of neurodivergence: qualitative variations in patterns of communication; difficulties with reciprocal social interactions; and a restricted, repetitive collection of behaviors and interests. For childhood autism, symptoms within all three areas of neurodivergence must be present before the age of 3 years.

When a child is suspected of having autism in Scania, they are referred to the Departments of Child and Adolescent Psychiatry and are examined by a multidisciplinary team^42^. These evaluations utilize both the Autism Diagnostic Observation Schedule-Generic (ADOS-G)^43^ and the Autism Diagnostic Interview-Revised (ADI-R)^44^ for most (75%) cases. Diagnostic methods for the remaining cases can differ. On occasion, structured instruments other than ADOS and ADI, such as the Mini-International Neuropsychiatric Interview for Children and Adolescents (MINI Kid), the Kiddie Schedule for Affective Disorders and Schizophrenia (K-SADS), the Social Communication Questionnaire (SCQ), the Social Responsiveness Scale, 2nd Edition (SRS-2), the Child Behavior Checklist (CBCL), or the Nordic Questionnaire for Evaluation of Development and Behavior in Children and Adolescents called Five-to-Fifteen (5–15 or FTF), are used to varying degrees and in varying combinations. In all cases, however, the child’s behavior is observed, the parents are interviewed, and information is gathered from the child’s school. All data is then evaluated and compared to diagnostic criteria in the Diagnostic and Statistical Manual of Mental Disorders (DSM). Finally, an autism diagnosis is assigned according to the ICD-10 and entered into the Skåne Healthcare Database (SHR). The outcome data used in this study was extracted from SHR and was available through the 31st of December, 2017.

### Covariates

The risk factors for autism development, identified a priori, considered for this study were maternal age (≤ 19, 20–34, ≥ 35), parity (1, 2, 3, or ≥ 4), pre-pregnancy body mass index (BMI; < 18.5, 18.5–24.9, 25–29.9, ≥ 30 kg/m^2^), smoking status at first antenatal visit (non-smoker, 1–9 cigarettes/day, ≥ 10 cigarettes/day), as well as sex of the child (male/female) and birth year (categorical; 2000–2009). Socioeconomic status (SES) predictors were also incorporated to control for potential confounding. These included maternal birth country (Sweden, Europe, other), maternal education (≤ 9, 10–12, 13–16, and > 16 years of schooling), annual household disposable income (quartiles), and neighborhood-level SES. Neighborhood-level SES was a continuous measure and captures the proportion of inhabitants in the neighborhood with “low economic standard”, which is defined by Statistics Sweden as the number of people living in a household with an economic standard that is less than 60% of the national median value^45^. Other covariates considered in sensitivity analyses included birth month and low birth weight.

### Statistical methods

The main analyses applied binary logistic regression models with ASD or childhood autism as the outcome in a univariate model and two multivariate models using exposure data during pregnancy where missing observations were replaced with imputed data. Concerning missing outcome and covariate data, however, complete-case analysis was applied. The first multivariate model was partially adjusted and included only variables that were significantly associated with the outcome when using the all-source (local) PM_2.5_ exposure variable: parity, pre-pregnancy BMI, sex of the child, and birth year. In addition to these variables, the second multivariate model, hereafter referred to as the fully adjusted model, also included maternal age, smoking status at first antenatal visit, maternal birth country, maternal education, annual household disposable income, and neighborhood-level SES. As correlation coefficients between the different sources of PM_2.5_ were very high (Supplementary Table S1, Supplementary Information), a single pollutant model was used. Linear exposure trends were assessed using a continuous increment corresponding to an interquartile range (IQR) increase in local PM_2.5_ concentrations. The PM_2.5_ sources' respective IQRs were as follows: 0.99 µg/m^3^ for all-source PM_2.5_, 0.33 µg/m^3^ for small-scale residential heating, 0.12 µg/m^3^ for tailpipe exhaust, and 0.31 µg/m^3^ for vehicle wear-and-tear. To check the assumption of linearity, associations were also investigated with the exposure variables in tertiles.

Three sensitivity analyses were conducted using the fully adjusted model with all-source PM_2.5_ as the exposure and childhood autism as the outcome. One adjusted for birth month as a categorical variable, as air pollution may vary substantially throughout the year; there may, therefore, be seasonal effects in the risk of autism^46^. The second excluded children with a low birth weight (LBW; < 2500 g) because birth weight is a potential mediator of the effect: air pollution increases the risk for low birth weight^47^, and low birth weight is a risk factor for autism^48^. Lastly, the children of mothers born outside of Sweden were excluded, as previous studies have shown much a higher risk of childhood autism among children whose mothers emigrated from non-European countries^47^.

All statistical analyses were carried out using SPSS version 27. Odds ratios (OR) and their corresponding 95% confidence intervals (CI) were reported for all analyses.

### Ethical considerations

The Lund University Ethical Committee approved this study prior to its realization (permission number 2014/696 and amendment 2016/211). As this was a register-based study, no formal informed consent was required. Lund University’s rules and protocols on data collection and processing were strictly followed. To ensure data integrity and safeguard confidentiality, sensitive data was stored in LUSEC, a highly secure data management platform hosted by the Faculty of Medicine, Lund University, Sweden, which utilizes data encryption and two-factor authentication. All methods were carried out in accordance with relevant guidelines and regulations. No personal identifying information is included in the present article.

## Results

Descriptive information on the exposures, covariates, and ASD is documented in Table 1. Relationships between ASD and various markers of socioeconomic status, smoking intensity, BMI, and PM_2.5_ exposure were seen.

**Table 1:** Mean and standard deviation (SD) of local PM_2.5_ concentrations (µg/m^3^) by source during pregnancy for autism spectrum disorder (ASD) and covariates.

	Number of subjects	All-source PM_2.5_	Small-scale residential heating	Tailpipe exhaust	Vehicle wear-and-tear	ASD* (%)
N	40,245	40,245	40,234	40,234	40,234	368 (0.9)
Total	40,245^†^	1.41 (0.64)	0.48 (0.25)	0.13 (0.08)	0.32 (0.21)	368 (0.9)
ASD						
No	39,877	1.41 (0.64)	0.48 (0.25)	0.13 (0.08)	0.32 (0.21)	–
Yes	368	1.54 (0.65)	0.53 (0.27)	0.15 (0.08)	0.36 (0.23)	–
Childhood autism						
No	39,877	1.41 (0.64)	0.48 (0.25)	0.13 (0.08)	0.32 (0.21)	0 (0)
Yes	282	1.55 (0.64)	0.51 (0.26)	0.15 (0.08)	0.38 (0.23)	282 (100)
Missing	86	1.51 (0.67)	0.60 (0.28)	0.14 (0.08)	0.31 (0.20)	86 (100)
Sex of the child						
Male	20,755	1.41 (0.63)	0.48 (0.26)	0.13 (0.08)	0.32 (0.21)	280 (1.3)
Female	19,490	1.41 (0.64)	0.48 (0.25)	0.13 (0.08)	0.32 (0.21)	88 (0.5)
Household disposable income level (quartiles)^‡^						
Lowest	9539	1.72 (0.63)	0.57 (0.28)	0.17 (0.08)	0.42 (0.22)	112 (1.2)
Lower middle	9910	1.52 (0.65)	0.53 (0.26)	0.14 (0.08)	0.35 (0.22)	104 (1.0)
Higher middle	10,733	1.25 (0.58)	0.44 (0.23)	0.11 (0.07)	0.27 (0.20)	87 (0.8)
Highest	10,050	1.17 (0.52)	0.40 (0.20)	0.11 (0.06)	0.25 (0.17)	65 (0.6)
Missing	13	1.20 (0.67)	0.38 (0.24)	0.12 (0.08)	0.30 (0.20)	0 (0)
Maternal education (years)						
≤ 9	4965	1.67 (0.63)	0.55 (0.27)	0.16 (0.08)	0.41 (0.22)	50 (1.0)
10–12	16,854	1.35 (0.64)	0.47 (0.25)	0.13 (0.08)	0.30 (0.21)	155 (0.9)
13–16	16,224	1.34 (0.60)	0.47 (0.24)	0.12 (0.07)	0.30 (0.20)	138 (0.9)
> 16	652	1.30 (0.51)	0.48 (0.22)	0.11 (0.06)	0.28 (0.16)	10 (1.5)
Missing	1550	1.86 (0.55)	0.58 (0.28)	0.19 (0.07)	0.49 (0.20)	12 (0.8)
Maternal age						
≤ 30	21,688	1.46 (0.65)	0.59 (0.25)	0.14 (0.08)	0.34 (0.22)	208 (1)
31–34	10,904	1.34 (0.61)	0.47 (0.24)	0.13 (0.07)	0.30 (0.20)	84 (0.8)
≥ 35	7653	1.37 (0.62)	0.48 (0.26)	0.13 (0.07)	0.31 (0.20)	76 (1.0)
Maternal smoking						
Non-smoker	34,193	1.40 (0.63)	0.48 (0.25)	0.13 (0.08)	0.32 (0.21)	302 (0.9)
1–9 cig/day	2676	1.47 (0.71)	0.51 (0.26)	0.14 (0.08)	0.33 (0.21)	30 (1.1)
≥ 10 cig/day	1091	1.51 (0.68)	0.53 (0.27)	0.14 (0.08)	0.34 (0.22)	14 (1.3)
Missing	2285	1.34 (0.62)	0.51 (0.25)	0.12 (0.07)	0.28 (0.19)	21 (0.9)
Maternal BMI (kg/m^2^)						
< 18.5	953	1.50 (0.64)	0.51 (0.25)	0.14 (0.08)	0.35 (0.22)	14 (1.5)
18.5 ≤ 25	22,444	1.41 (0.63)	0.48 (0.24)	0.13 (0.08)	0.32 (0.21)	174 (0.8)
25 ≤ 30	8731	1.41 (0.65)	0.48 (0.26)	0.13 (0.08)	0.32 (0.22)	94 (1.1)
≥ 30	3732	1.42 (0.68)	0.48 (0.28)	0.14 (0.08)	0.33 (0.23)	51 (1.4)
Missing	4385	1.35 (0.62)	0.50 (0.24)	0.12 (0.07)	0.29 (0.19)	34 (0.8)
Maternal birth country						
Sweden	28,088	1.26 (0.60)	0.45 (0.24)	0.12 (0.07)	0.27 (0.19)	257 (0.9)
Europe	5326	1.65 (0.61)	0.53 (0.26)	0.16 (0.07)	0.41 (0.21)	53 (1.0)
Other	6830	1.83 (0.53)	0.58 (0.27)	0.18 (0.07)	0.47 (0.20)	58 (0.8)
Parity						
1st child	19,241	1.44 (0.64)	0.49 (0.25)	0.14 (0.08)	0.34 (0.22)	207 (1.1)
2nd child	13,396	1.33 (0.61)	0.47 (0.25)	0.12 (0.07)	0.29 (0.20)	98 (0.7)
3rd child	4973	1.37 (0.64)	0.48 (0.25)	0.13 (0.08)	0.31 (0.20)	47 (0.9)
≥ 4th child	2635	1.62 (0.66)	0.55 (0.28)	0.16 (0.08)	0.39 (0.23)	16 (0.6)
Birth year						
2000	3745	1.56 (0.69)	0.71 (0.30)	0.15 (0.08)	0.28 (0.17)	32 (0.9)
2001	3912	1.50 (0.65)	0.66 (0.26)	0.14 (0.08)	0.29 (0.18)	43 (1.1)
2002	4379	1.55 (0.68)	0.66 (0.26)	0.15 (0.08)	0.31 (0.19)	51 (1.2)
2003	4380	1.46 (0.65)	0.57 (0.23)	0.14 (0.08)	0.30 (0.20)	45 (1.0)
2004	4509	1.38 (0.63)	0.47 (0.18)	0.13 (0.07)	0.30 (0.20)	46 (1.0)
2005	4689	1.27 (0.58)	0.38 (0.14)	0.12 (0.07)	0.30 (0.21)	33 (0.7)
2006	4074	1.31 (0.59)	0.33 (0.12)	0.12 (0.07)	0.35 (0.23)	40 (1.0)
2007	4557	1.33 (0.64)	0.32 (0.12)	0.13 (0.07)	0.36 (0.24)	35 (0.8)
2008	4601	1.42 (0.64)	0.38 (0.19)	0.14 (0.08)	0.39 (0.26)	31 (0.7)
2009	1399	1.22 (0.54)	0.24 (0.11)	0.11 (0.97)	0.34 (0.23)	12 (0.9)

*PM*_*2.5*_ particulate matter with a diameter of < 2.5 µm, *Cig* cigarette, *BMI* body mass index.

*International Classification of Mental and Behavioral Disorders version 10 (ICD-10) diagnoses codes beginning with F84.

^†^Given for all-source PM_2.5_ (N may vary slightly by PM_2.5_ source).

^‡^Measured as annual disposable income. A category of missing is given for variables where more than one observation has missing data.

All-source PM_2.5_ exposure during pregnancy was associated with ASD, with ORs of 1.33 (95% CI 1.13–1.57) in the partially adjusted model and 1.22 (95% CI 0.99–1.50) in the fully adjusted model (Table 2). An association was also found for exposure to PM_2.5_ from small-scale residential heating and ASD in the partially adjusted model [1.22 (95% CI 1.08–1.37)], but the association was less pronounced in the fully adjusted model: 1.12 (95% CI 0.94–1.34). Regarding the road traffic-related sources, both tailpipe exhaust and vehicle wear-and-tear were associated with ASD: ORs in the fully adjusted model were 1.30 (95% CI 1.05–1.60) and 1.24 (95% CI 1.02–1.50), respectively. Analyses utilizing exposure variables in tertiles did not suggest any major deviations from linearity (data not shown).

**Table 2:** Odds ratios (OR) and their 95% confidence intervals (CI) for autism spectrum disorders (ASD; ICD-10 codes starting with F84) associated with an interquartile range (IQR) increase in concentrations (µg/m^3^) of the investigated sources of local PM_2.5_ during pregnancy.

	Crude model		Partially adjusted model^†^		Fully adjusted model^‡^	
	OR	(95% CI)	OR	(95% CI)	OR	(95% CI)
All-source PM_2.5_	1.36	(1.17–1.58)	1.33	(1.13–1.57)	1.22	(0.99–1.50)
Small-scale residential heating	1.22	(1.10–1.34)	1.22	(1.08–1.37)	1.12	(0.94–1.34)
Tailpipe exhaust	1.38	(1.19–1.60)	1.36	(1.16–1.59)	1.30	(1.05–1.60)
Vehicle wear-and-tear	1.29	(1.12–1.48)	1.32	(1.14–1.54)	1.24	(1.02–1.50)

IQRs: all-source PM_2.5_ = 0.99 µg/m^3^, small-scale residential heating = 0.33 µg/m^3^, tailpipe exhaust = 0.12 µg/m^3^, vehicle wear-and-tear = 0.31 µg/m^3^.

^†^Adjusted for maternal parity, pre-pregnancy BMI, sex of the child, and birth year.

^‡^Adjusted for all covariates included in the partially adjusted model as well as maternal age, smoking status, birth country, education, income, and neighborhood-level SES. Number (N) of women included in each model (given for all-source PM_2.5_; N may vary slightly by PM_2.5_ source): Crude model = 40,245 (number of cases = 368), Partially adjusted model = 35,860, Fully adjusted model = 29,383.

Results for childhood autism (Table 3) were somewhat more pronounced compared to ASD as a group, with ORs of 1.34 (95% CI 1.05–1.70) for all-source PM_2.5_, 1.18 (95% CI 1.01–1.39) for small-scale residential heating, 1.46 (95% CI 1.15–1.85) for tailpipe exhaust, and 1.36 (95% CI 1.10–1.68) for vehicle wear-and-tear in fully adjusted models.

**Table 3:** Odds ratios (OR) and their 95% confidence intervals (CI) for childhood autism (ICD-10 code F84.0) associated with an interquartile range (IQR) increase in concentrations (µg/m^3^) of the investigated sources of local PM_2.5_ during pregnancy.

	Crude model		Partially adjusted model^†^		Fully adjusted model^‡^	
	OR	(95% CI)	OR	(95% CI)	OR	(95% CI)
All-source PM_2.5_	1.39	(1.17–1.65)	1.25	(1.11–1.42)	1.34	(1.05–1.70)
Small-scale residential heating	1.15	(1.01–1.30)	1.25	(1.11–1.42)	1.18	(1.01–1.39)
Tailpipe exhaust	1.44	(1.22–1.70)	1.50	(1.26–1.80)	1.46	(1.15–1.85)
Vehicle wear-and-tear	1.41	(1.21–1.65)	1.43	(1.21–1.69)	1.36	(1.10–1.68)

IQRs: all-source PM_2.5_ = 0.99 µg/m^3^, small-scale residential heating = 0.33 µg/m^3^, tailpipe exhaust = 0.12 µg/m^3^, vehicle wear-and-tear = 0.31 µg/m^3^.

^†^Adjusted for maternal parity, pre-pregnancy BMI, sex of the child, and birth year.

^‡^Adjusted for all covariates included in the partially adjusted model as well as maternal age, smoking status, birth country, education, income, and neighborhood-level SES. Number (N) of women included in each model (given for all-source PM_2.5_; N may vary slightly by PM_2.5_ source): Crude model = 40,159 (number of cases = 282), Partially adjusted model = 35,764, Fully adjusted model = 29,313.

Concerning the sensitivity analyses using the fully adjusted model, adjusting for birth month did not affect the OR estimate for childhood autism: 1.34 (95% CI 1.06–1.70). Additionally, when excluding LBW babies, the OR for childhood autism was 1.35 (95% CI 1.06–1.72), which is similar to the OR for all births [1.34 (95% CI 1.05–1.70)]. Limiting the study population to Swedish-born women resulted in lower ORs compared to the entire study population. For example, the OR for childhood autism associated with an IQR increase in all-source PM_2.5_ was 1.16 (95% CI 0.87–1.54) among Swedish-born women, compared to 1.34 (95% CI 1.05–1.70) for all women.

## Discussion

### Main findings

In this population-based study from southern Sweden, associations were observed between exposure to nearly all investigated sources of local PM_2.5_, except for small-scale residential heating, during pregnancy and the broad diagnosis group ASD. Exposure to locally produced PM_2.5_ from each investigated source (all-source PM_2.5_, small-scale residential heating, tailpipe exhaust, and vehicle wear-and-tear) was associated with childhood autism, with associations being somewhat more pronounced than for ASD. Because ASD comprises all pervasive developmental disorders for which symptoms of neurodivergence do not have to be present by a specified age, these results may reflect the larger heterogeneity within the ASD group compared to childhood autism, which has stricter, more uniform diagnostic criteria.

When considering only mothers born in Sweden, the associations tended to be lower than for that of the entire study population. Findings from previous research on environmental injustice in Scania showed that non-Swedish-born persons, as well as those with less education and lower income, had higher odds of being exposed to greater concentrations of air pollution^49^. In Sweden, children born to women who emigrated from Sub-Saharan Africa and the Middle East are, furthermore, more commonly diagnosed with autism^47^. Other than air pollution exposure and SES risk factors, a possible explanation for the higher proportion of autism diagnoses among immigrants is, for example, vitamin D deficiency^50^, which is particularly predominant among immigrants from Sub-Saharan Africa and the Middle East living in Scandinavia^51–53^. Although data on vitamin D deficiency was not available for the present study, it is unlikely that this would explain our findings, especially since controlling for birth month did not affect the results.

### Small-scale residential heating

No previous epidemiological studies investigating the effects of source-specific ambient wood smoke exposure from small-scale residential heating on autism development could be identified. Concerning neurological conditions in general, a longitudinal study in northern Sweden has indicated that PM_2.5_ from residential wood burning is associated with dementia incidence^12^. In an experimental study, exposure to wood smoke particles induced cytotoxicity and disrupted proliferation in exposed first trimester placenta cells; particles detected inside the cells also caused structural damage to mitochondria and endoplasmic reticulum^54^. Existing source-apportionment studies investigating prenatal exposures have mainly included pregnancy complications, where Delta-C (a marker for wood smoke) during wintertime was found to be associated with greater odds of developing early-onset preeclampsia^55^, and birth outcomes, where PM_2.5_ from biomass burning (i.e., ambient wood smoke) was associated with a lower risk of preterm birth^56^, low birth weight^57^, and stillbirth^58^. Despite this evidence on other adverse health effects, additional studies on wood smoke and autism are needed to corroborate our results.

Due to its unique chemical composition, PM_2.5_ derived from wood smoke may have varying toxicity compared to other sources of ambient PM_2.5_. For example, a 2003 review stated that studies including residential wood combustion as a major source of PM reported higher relative risks for adverse health outcomes compared to general ambient PM estimations^59^. A study examining short-term exposure also found higher statistically significant risk-increases in mortality when 24-h average concentrations were used and stronger increased risks for deaths occurring in the cold season, both of which better represent PM_2.5_ exposure from wood burning compared to traffic^60^. There is much heterogeneity in the literature surrounding this source, its origins and its assessment, however, as a 2018 review found ambient PM from biomass burning to be less detrimental than other sources^33^, with a noted exception from Copenhagen^61^. In the present study, emphasis is placed on the positive associations observed for all PM_2.5_ sources, including small-scale residential heating, as opposed to comparing the size of each source's individual point estimates. Regardless, residential wood burning remains an air quality concern. Initiatives aiming to reduce ambient PM_2.5_ and PM_10_ concentrations from wood smoke have luckily proven successful and have been shown to improve public health^62^.

### Road traffic-related sources

Our results suggest that both local PM_2.5_ from tailpipe exhaust and vehicle wear-and-tear contribute to the observed associations with autism. These findings are in line with our previous study on prenatal exposure to ambient NO_X_ concentrations, mainly from traffic, and autism using the same cohort (MAPSS), where children in the highest exposure quartile had a 40% greater risk of developing ASD compared to those in the lowest^42^. Under the assumption of a causal association between PM_2.5_ and childhood autism, a health impact assessment conducted in Scania identified 3% of autism cases to be attributable to locally emitted PM_2.5_, of which ~ 30% is derived from traffic^63^. Outside our study setting, two case–control studies from California found 15% increased odds^64^ and just over double the risk^65^ of autism development due to traffic-related PM_2.5_ exposure during pregnancy.

However, studies conducted in Stockholm, Sweden, did not find associations between exposure to traffic-related air pollution during pregnancy and ASD^66,67^. Reasons for this conflict may include that those studies considered PM_10_ and NO_X_, while ours investigated PM_2.5_, which is the pollutant with the strongest evidence in connection to autism development according to systematic reviews^18,19^. Another register-based study in Stockholm not observing associations used “symptoms of neurodevelopmental disorders” as opposed to physician-diagnosed ASD^66^. Similarly, a study using four European cohorts, including a Swedish one, explored autistic traits, but did not find an association with air pollution, even for PM_2.5_; here, land-use regression models, with predictor variables including traffic and space heating, were used^68^. In neighboring Denmark, researchers found that exposure to traffic-related NO_2_, PM_10_, and PM_2.5_ in early infancy, not during pregnancy, was associated with autism^69^. In the present study, exposure during fetal life and postnatal life could not be adequately differentiated.

One study, conducted in Southern California, USA, on prenatal exposure to traffic-related PM_2.5_ that further differentiated between tailpipe exhaust and vehicle wear-and-tear in association with autism was identified^32^. Authors used tracers of carbonaceous tailpipe PM_2.5_, including elemental carbon (EC) and organic carbon (OC), from diesel and gasoline fuel combustion as well as non-tailpipe particles rich in trace metals (copper, iron and manganese) from re-suspended dust, tire-wear and brake-wear. The observed hazards ratios (HR) for tailpipe tracers of PM_2.5_ were 1.11 (95% CI 1.06–1.16), 1.09 (95% CI 1.04–1.15) per interquartile increase of EC and OC, respectively^32^. For interquartile increases in non-tailpipe tracers of copper, iron and manganese, the HRs were 1.09 (95% CI 1.04–1.13), 1.14 (95% CI 1.09–1.20), and 1.17 (95% CI 1.12–1.22), respectively^32^. This is in line with our findings that PM_2.5_ from both tailpipe exhaust and vehicle wear-and-tear may contribute to air pollution’s association with ASD. Interestingly, Rahman and colleagues found that the associations for tailpipe tracers were considerably attenuated when analyses were adjusted for non-tailpipe sources, while estimates for non-tailpipe tracers were generally unaltered when adjusting for tailpipe sources^32^. This indicates that the HRs for non-tailpipe emissions and ASD were independent of the effects of tailpipe emissions, further supporting their importance as a traffic-related source with negative health impacts.

### Future research

Systematic reviews on air pollution exposure and ASD development have noted both the pregnancy and postnatal periods to be decisive exposure windows^19^. With this, future studies should prioritize the discernment and investigation of specific vulnerable periods, such as pregnancy trimesters and late gestation versus the first year(s) of life, which was not possible in the present study.

The continued inclusion of residential wood burning in source-specific air pollution epidemiology is pertinent, as it has been shown to be a significant source of ambient PM, especially in wintertime, and has also been identified as a potential challenge toward air quality control due to the increase in recreational wood stove-use, particularly in urban areas across Europe^70^. Moreover, recent reviews^33,35^ on the health effects of PM exposure attributable to wood combustion are limited to mainly respiratory outcomes, with some cardiovascular and oncological outcomes included. Future research should, therefore, consider investigating additional health effects^35^, such as the development of autism where current literature is lacking. Such source-apportionment studies are needed to substantiate our results.

As only one prior study on autism could be identified that apportioned PM_2.5_ road traffic emissions into their respective tailpipe exhaust and vehicle wear-and-tear sources, future studies should consider source-specific separation. This source-apportionment of traffic-related air pollution would also be valuable for research on other health outcomes. The subsequent findings would provide a greater understanding of how distinct PM_2.5_ sources affect human health. Importantly, vehicle wear-and-tear from re-suspended dust, tires and brakes will continue to be a relevant source of PM, especially as larger proportions of vehicle fleets become electrified and tailpipe emissions are reduced.

### Methodological considerations

A key strength of this study is its large sample population derived from the MAPSS birth cohort, comprising 98% of all births occurring in the hospital catchment areas throughout Scania. Utilizing a validated, high resolution dispersion model to estimate PM_2.5_ exposure and obtaining the exact geographic coordinates of each woman’s home residence were also vital for accurate exposure estimates at the individual level. An additional strength includes the thorough outcome assessment performed by Departments of Child and Adolescent Psychiatry. It should be mentioned, however, that the prerequisites for autism diagnosis have likely changed over time worldwide. For instance, a Swedish study reported that the presence of significantly fewer symptoms appeared to be necessary to receive a clinical autism diagnosis between the years 2004 and 2014 compared to 1998 and 2007^71^. Any such trends in our data should not have affected the results because the analyses were adjusted for birth year.

Furthermore, health care systems in Sweden are tax-subsidized and are used by virtually all residents, which increases the ability to identify physician-diagnosed cases of ASD and childhood autism and record them in high quality healthcare databases. With this, outcome misclassification, response-bias, recall-bias, and selection bias were likely avoided. Information on covariates were similarly collected from well-managed, precise registers. Our results are also considered generalizable to study areas where the populations and sources of air pollution are comparable. The findings are relevant to both public health in general and the clinical setting specifically because they indicate that locally produced PM_2.5_ concentrations can affect the risk of autism among children. Finally, this study contributes evidence to an emerging research area investigating the health effects of local, source-specific air pollution exposure. Interestingly, accumulating evidence suggests that locally produced PM may be more hazardous to human health than regional, background concentrations^72^.

This study also has several limitations. A sizeable proportion of the study population was missing data on exposure, outcome, and/or covariates. While missing exposure values were able to be imputed using exposure data from subsequent years, the absence of detailedness in the exposure data hindered our ability to fully distinguish between sensitive periods of pregnancy, i.e., trimesters, as well as between the entire pregnancy period and the first year(s) of life. These exposure windows tend to be highly correlated because air pollution concentrations are relatively stable between years, and only a limited proportion of women move to a new residential address, with new exposure concentrations, directly after giving birth. Still, the present study aims to describe prenatal exposure, and it is, therefore, described as such. Another limitation is that data on parental diagnoses was not available. Given that genetic factors account for a considerable part of the variation in autism development and emergence^73^, our results could partly be explained by heredity, if parents with autism were more likely to reside in areas characterized by higher concentrations of air pollution than parents without autism. Residual confounding may be present due to other risk factors for autism that are also associated with the exposure but are not accounted for in our statistical models, such as lifestyle factors and vitamin D deficiency. There is also potential for residual confounding with respect to SES, as indicated by results from the sensitivity analysis on Swedish-born women. Exposure misclassification may exist, as exposure was assessed at each woman’s home residence, and participants’ total exposure, including indoor, behavior-related, transport-related, and occupational, was not considered. However, this approach is deemed standard practice in air pollution epidemiology research, with the assumption that the resulting misclassification is non-differential. Moreover, we could not clearly distinguish between tailpipe exhaust and vehicle wear-and-tear due these sources’ high correlation (Supplementary Table S1, Supplementary Information). Instead, more studies, preferably in a multi-cohort setting, are needed to increase statistical power. Finally, because the differences in ORs between the investigated sources of local PM_2.5_ were not formally tested, direct comparisons between our source-specific risk estimates could not be made.

## Conclusion

These findings add to current evidence that prenatal exposure to ambient air pollution is associated with an increased risk of developing ASD, particularly childhood autism, and offers insight into these associations in a relatively low exposure setting. Additionally, this source-specific study indicates that both local PM_2.5_ from small-scale residential heating (mainly wood burning) and road traffic (tailpipe emissions and vehicle wear-and-tear) are relevant exposure sources. Our results also support existing literature that has documented the substantial health effects of locally produced particles, despite their relatively small contribution to PM’s total concentrations compared to long-range, in-transported particles.

## Supplementary Information

Supplementary Information.

## Data Availability

The datasets generated during and/or analyzed during the current study are stored on a secure server and are not publicly available because they contain sensitive information (on health data, demographic characteristics, socioeconomic status) and, therefore, cannot be shared openly. However, they are available from Anna Oudin (anna.oudin@med.lu.se) on reasonable request.
