# Supplementary material for: Exposure to local, source-specific ambient air pollution during pregnancy and autism in children: a cohort study from southern Sweden
Source: Sci Rep. 2023 Mar 8;13:3848. doi: 10.1038/s41598-023-30877-5 (PMC9995328; doi:10.1038/s41598-023-30877-5)
Supplement: Supplementary file 1 — Supplementary Information. [file 41598_2023_30877_MOESM1_ESM.pdf]

Supplementary Information

Supplementary Figure S1. Boxplot graphs describing the concentration distributions (µg/m^3) of locally produced $PM_{2.5}$ from **A)** all-source $PM_{2.5}$, **B)** small-scale residential heating, **C)** tailpipe exhaust, and **D)** vehicle wear-and-tear during pregnancy throughout Scania, Sweden.

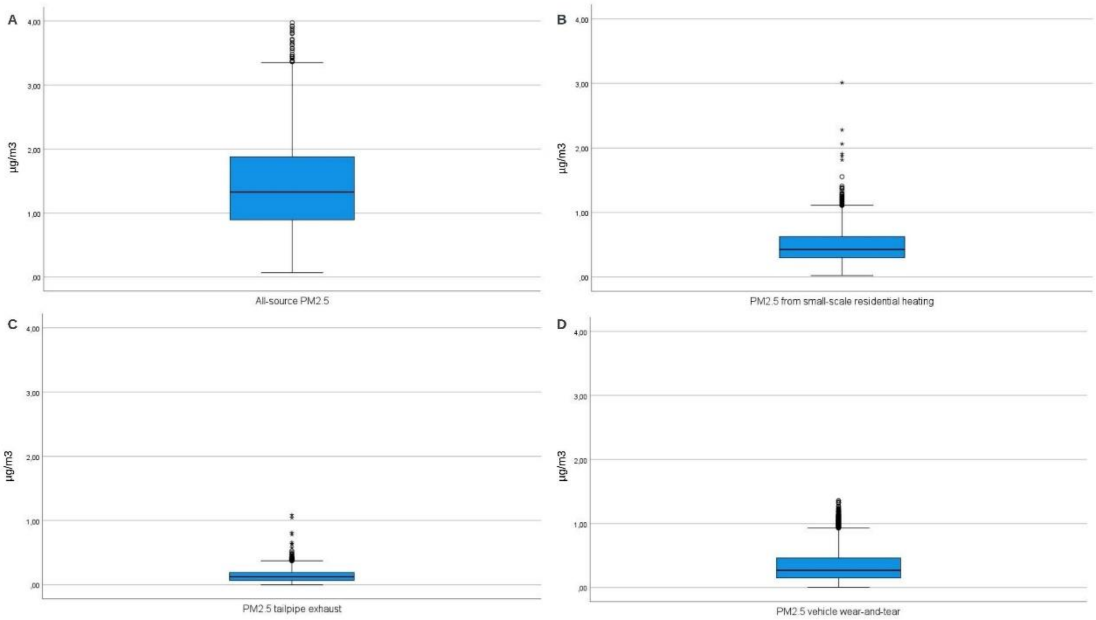

Supplementary Table S1. Pearson correlation coefficients between the investigated sources of local $PM_{2.5}$ during pregnancy. All p-values were <0.001.

	All-source $PM_{2.5}$	Small-scale residential heating	Tailpipe exhaust	Vehicle wear-and-tear
All-source $PM_{2.5}$	1	0.76	0.91	0.92
Small-scale residential heating		1	0.63	0.55
Tailpipe exhaust			1	0.95
Vehicle wear-and-tear				1
